# Combining radiological and radiation oncology expertise in the delineation of hypopharyngeal tumours: potential effects on treatment volumes and patterns of failure

**DOI:** 10.1016/j.ctro.2025.101022

**Published:** 2025-07-24

**Authors:** Gabriella Alexandersson von Döbeln, Eva Onjukka, Halla Sif Ólafsdóttir, Sara Jonmarker Jaraj, Mattias Hedman

**Affiliations:** a Department of Radiation Oncology, Karolinska University Hospital, 171 76 Stockholm, Sweden; b Division of Surgery, Department of Clinical Science, Intervention and Technology, Karolinska Institutet, SE-141 52 Huddinge, Sweden; c Department of Nuclear Medicine and Medical Physics, Karolinska University Hospital, 171 76 Stockholm, Sweden; d Department of Oncology-Pathology, Karolinska Institutet, 171 77 Stockholm, Sweden; e Department of Neuroradiology, Karolinska University Hospital, 171 76 Stockholm, Sweden

**Keywords:** Radiotherapy, Hypopharyngeal cancer, Target definition, Outcome

## Abstract

**Background and purpose:**

Target definition is one of the greatest uncertainties in the radiotherapy process. We aimed to investigate whether a radiologist specialized in head and neck can improve the target definition of hypopharyngeal cancers.

**Materials and methods:**

We retrospectively identified 54 patients with hypopharyngeal cancer who received curative-intent radiotherapy between 2009–2015. New target structures were defined incorporating head and neck radiology expertise and updated delineation guidelines. The new structures were subsequently compared both quantitively and qualitatively to the original delineations. Loco-regional failures were analyzed in relation to radiotherapy dose and target volumes.

**Results:**

There was a significant reduction in gross tumour volume (GTV) for the primary tumour, decreasing from 14.4 to 9.2 cm^3^ (−47 %), and in clinical target volume (CTV), decreasing from 203.7 to 93.8 cm^3^ (−54 %). Mean quantitative values indicated a large overestimation of the original GTV (Dice Coefficient 0.58 ± 0.2 SD, Jaccard index 0.44 ± 0.19 SD, Positive predictive value 0.53 ± 0.24 SD). Only 39 % of the original primary tumour GTV and 19 % of the original lymph node GTV were assessed as acceptable. Twelve patients (22 %) had a locoregional recurrence. In relation to both the original radiation dose and the updated dose distribution, nine recurrences were classified as in field, two as marginal, and one could not be evaluated. The 3-year and 5-year locoregional progression free survival (PFS) was 75.5 % and 66.6 % respectively.

**Conclusion:**

Incorporating radiological expertise in the delineation of hypopharyngeal tumours leads to large changes in tumour volumes and possibly a decrease in radiation volumes which may lead to reduced side effects.

## Highlights

• A collaboration between a radiologist and a radiation oncologist is feasible when delineating tumour and target volumes.
• There is a reduction of GTV volumes when a radiologist is included in defining the target for hypopharyngeal cancers.
• A low concordance between the original and the new GTVp and GTVn, indicates a significant impact of the radiologist.

## 1 Introduction

Hypopharyngeal cancer is associated with the worst prognosis of all cancers in the head and neck region, with a 5-year survival of 27 % in Sweden [1]. Most patients are diagnosed when the tumour is not resectable. For patients with good performance status, the treatment of choice is then radiotherapy combined with chemotherapy. Upfront surgery is reserved for a minority of patients with limited tumour extension. Despite improvements in radiotherapy techniques, the outcome has remained poor, even for patients with localized disease. Around 50 % of patients experience a recurrence within the first year after treatment, as a locoregional recurrence or with peripheral metastases. The locoregional failures are most often seen in the areas irradiated with full radiation dose (68–70 Gy) [2] which has also been demonstrated in other head and neck cancers [3].

Delineation of gross tumor volume (GTV) and clinical target volume (CTV) remains one of the greatest sources of uncertainty when treating patients with radiotherapy. This issue has been addressed by introducing guidelines and peer-review of delineations [[4], [5], [6]]. The risk of developing side effects during and after treatment [7], as well as the probability of tumour control, is strongly related to the treatment volume. A correct target definition is essential to enable dose escalation for better tumour control and to avoid unnecessary irradiation, thereby minimizing side effects.

In the current study, we aimed to retrospectively evaluate the impact of including a radiologist specialized in head and neck alongside a radiation oncologist when defining the GTV. A secondary aim was to analyze CTV volumes over time and the patterns of failure in relation to the delineated volumes and the delivered radiotherapy.

## 2 Material/Methods

### 2.1 Study design

We conducted a retrospective, single-center cohort study.

### 2.2 Study population

Patients included were treated at Karolinska University Hospital in Stockholm, Sweden between January 1st, 2009, and December 31st, 2015. They were referred to the radiotherapy department from the head and neck surgical department following treatment recommendations from the multidisciplinary tumour board in accordance with institutional standards. The patients had squamous cell carcinoma of the hypopharynx and were treated with definitive radiotherapy (>60 Gy, Equivalent Dose in 2-Gy fractions (EQD2) with α/β = 10 Gy) with or without concomitant cetuximab or chemotherapy and/or induction chemotherapy. The patients were identified in the ARIA Oncology Information System (OIS) (Varian medical systems, USA). From 2012, radiotherapy was administered using the Volumetric Arc Technique (VMAT). Before 2012, treatment was delivered with 3D conformal radiation therapy. The most common schedule was conventionally fractionated 68 Gy to the GTV and 46 Gy to elective areas, in 34 and 23 fractions respectively, according to national guidelines [1]. Some patients were treated with an accelerated regimen, 1.1 Gy + 2 Gy daily (minimum 6 h apart), 5 days a week for 4.5 weeks, total dose 68 Gy.

### 2.3 Clinical follow-up and data collection

Clinical follow-up was according to departmental standard. In general, clinical evaluations were performed every 3 months for the first two years, and then every 6 months for the following three years. The tumours were staged according to UICC, TNM classification of malignant tumours, 8th edition, 2017. In the current study, patient characteristics and recurrence location and time were collected by reviewing medical records.

### 2.4 Target delineation evaluation

The original GTV and CTV were defined by a radiation oncologist only and guided by diagnostic radiology and written description of diagnostic endoscopic examinations performed by a head and neck surgeon. Diagnostic procedures were done before the initiation of (chemo)-radiotherapy. All patients were peer-reviewed on an institutional round with senior radiation oncologists before treatment. For the purpose of this study, a head and neck radiologist, together with a radiation oncologist retrospectively and blinded to the original delineations, defined a new GTV for all patients, using the same radiology imaging and endoscopic descriptions as the original contours were based upon. New CTVs were subsequently defined by the radiation oncologist, and peer-reviewed by a second radiation oncologist, both with over ten years’ experience of working with head and neck cancer patients and using current guidelines implemented in our clinic after 2015 [[8], [9], [10]].

Any differences between the new and the original GTV and CTV delineations were evaluated both qualitatively and quantitatively. The same two radiation oncologists performed a qualitative comparison, grading the alignment as follows: no overlap (1), unacceptable deviation (2), acceptable deviation (3), good concordance (4). Several quantitative measures of the delineation concordance between the original and the new GTV and CTV delineations were calculated, using MICE toolkit (NONPIMedical AB, Sweden). These measures included the DICE coefficient [11], the Jaccard index (the intersection divided by the union), the mean Hausdorff distance (the mean distance for all point pairs on the new and old structure surfaces, respectively), the sensitivity (the fraction of the new volume that was included in the old volume), and the positive predictive value (the fraction of the old volume included in the new volume).

### 2.5 Definition of failure site

For patients with tumour recurrence, the first diagnostic CT scan visualizing recurrence was imported into ARIA. Rigid registration was used to assess the relation to the given radiotherapy and to new treatment plans based on the updated delineations. The localization of the recurrence on the original treatment-planning CT image was then indicated by a head and neck radiologist. The recurrences were subsequently identified as “in-field failure” if the recurrence was within the volume that received ≥95 % of dose, “marginal failure” if the recurrence was in the volume that received 20–95 % of the dose, and “out-of-field failure” if the recurrence was in a volume treated with less than 20 % of the prescribed dose.

### 2.6 Definition of survival measures

Locoregional PFS was defined as the time from the last day of the radiotherapy to the date of positive cytology, or the first radiological image demonstrating a recurrence if cytology was not performed. Overall survival (OS) was defined as the time in months from the last day of the radiotherapy to death from any cause. The last day of follow-up was June 1st, 2022, securing at least 75 months of follow-up for living patients. For patients lost to follow-up the last date of follow-up was their last appointment at the clinic.

### 2.7 Statistical analyses

The Kaplan-Meier approach was used to measure locoregional PFS and OS. Patients were censored at the date of death or at the end of follow-up.

A Spearman correlation analysis was performed between the qualitative and quantitative measures of the delineation concordance, to determine which quantitative measure might be the most relevant for the current application. Matlab version R2022A (Mathworks, USA) was used for this analysis.

All tests were 2-sided and statistical significance was considered with a p-value less than 0.05. The statistical analyses were performed using R version 3.6.1. (R basis for statistical calculation, Vienna University of Economics and Business, Vienna, Austria).

### 2.8 Ethical approval

All procedures were performed in compliance with relevant laws and institutional guidelines and this study was approved by the Swedish Ethical Review Authority, Dnr: 2019-03137.

## 3 Results

**Fig. 1:**
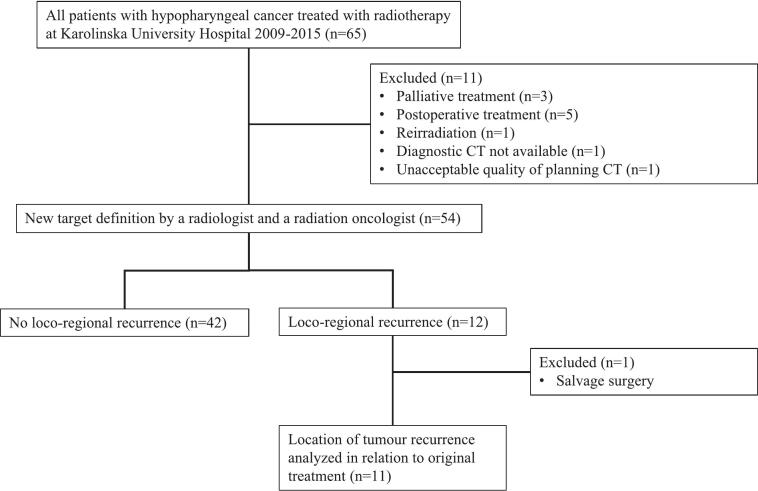
Flowchart of study measures.

Fifty-four patients were included in the analyses (Fig. 1). The majority of patients were men, and the median age at diagnosis was 68 years. All patients, except one, were current smokers or had a history of smoking. Most of the patients had a performance status of 0–1, according to the Eastern Cooperative Oncology Group Performance Status Scale. Comorbidity was graded according to the Charlson Comorbidity Index (omitting solid tumour in the calculation of the score), with a median index of three [12]. However, 24 % of the patients had an index of five or higher, indicating that a considerable number of patients had severe comorbidity at the time of treatment. The delivered median radiotherapy dose was 68 Gy (range 61–68 Gy). 43 patients received conventionally fractionated treatment, 39 with sequential and four with simultaneously integrated boost. Ten patients were treated with two fractions per day, six hours apart, 2 Gy and 1.1 Gy respectively. One patient received 16 Gy in 8 fractions followed by 45 Gy in 15 fractions. Twenty-eight patients were treated with VMAT while 26 patients were treated with 3D conformal technique. Thirty-eight patients were treated with induction chemotherapy and/or concomitant chemotherapy or cetuximab. 16 patients were treated exclusively with radiotherapy. Patient characteristics are shown in Table 1.

**Table 1:** Patient characteristics.

**Characteristics**	**no. (%)(N = 54)**

**Sex**	
Male	41 (76)
Female	13 (24)

**Age**	
Median yr	68
Range yr	42–85

**Performance status^1^**	
ECOG PS 0–1	45 (83)
ECOG PS 2	7 (13)
Unknown	2 (4)

**Charlson Comorbidity Index^2^**	
0–2	15 (28)
3–4	26 (48)
>5	13 (24)
Median CCI	3

**Smoking status^3^**	
Current smoker	36 (67)
Former smoker	17 (31)
Never smoker	1 (2)

**Stage according to TNM^4^**	
Stage I-II	7 (13)
Stage III	9 (17)
Stage IV	38 (70)

**Tumour stage**	
T1	2 (4)
T2	19 (35)
T3	16 (30)
T4	17 (31)

**Nodal stage**	
N0	15 (28)
N1	6 (11)
N2	33 (61)
N3	0 (0)

**Chemoterapy^5^**	
Yes	38 (70)
No	16 (30)
**Median dose (Gy, range)**	68 (61–68)

**Neck dissection^6^**	
Yes	15 (28)
No	39 (72)

1. According to the Eastern Cooperative Oncology Group Performance Status Scale.

2. Solid tumour omitted from the calculation.

3. Current smoker = current smoker or stopped within the last 12 months. Former smoker = stopped smoking since at least 12 months.

4. According to UICC, TNM classification of malignant tumours, 8th edition, 2017.

5. Induction or concomitant chemotherapy.

6. After oncological treatment.

Out of the 54 patients, three were lost to follow-up after a median time of 47 months.

After re-delineation, there was a significant decrease in the median GTVp and CTV volumes (Table 2). Qualitatively, 21 (39 %) of the original GTVp delineations and 10 (19 %) of the original GTVn delineations, were considered acceptable (graded 3 and 4) when compared to the structures jointly defined by the radiologist and the radiation oncologist.

**Table 2:** Original and updated GTV and CTV volumes.

Target volume	Original volume, cm^3^ (median, range)	Updated volume, cm^3^ (median, range)	Change (%)	p value
Primary tumour GTV (GTVp)	17.4 (0.9–100)	9.2 (0.9–76.4)	−47	0.04
Lymph node GTV (GTVn)	11.0 (0.8–121.1)	9.8 (0.6–143.9)	−11	0.67
Clinical tumour volume (CTV)	203.7 (33.4–677.5)	93.8 (21.7–417.9)	−54	<0.01

The quantitative measures of the delineation concordance, as shown in Table 3, indicate a large overestimation of the original GTV. The positive predictive value means that 59 % of the original GTVp and 74 % of the original GTVn consisted of the updated GTVs. The sensitivity value means that 78 % of the updated GTVp and 68 % of the updated GTVn were included in the original delineation.

**Table 3:** Quantitative concordance measures between the original and the updated structures.

	CTV (median, range)	GTVp (median, range)	GTVn (median, range)
Dice Coefficient	0.62 (0.26–0.85)	0.63 (0.01–0.86)	0.58 (0.02–0.90)
Jaccard index	0.45 (0.15–0.74)	0.46 (0.00–0.75)	0.41 (0.01–0.82)
Mean Hausdorff (mm)	2.47 (0.49–7.81)	1.13 (0.36–10.7)	1.25 (0.14–23.6)
Sensitivity	0.90 (0.52–1.00)	0.78 (0.01–0.96)	0.68 (0.04–0.97)
Positive predictive value	0.48 (0.15–0.94)	0.59 (0.01–0.90)	0.74 (0.01–0.99)

The quantitative measure that showed the strongest correlation with the qualitative score was sensitivity (Table 4), with statistical significance for CTV and GTVp. For GTVp, the mean Hausdorff distance, Dice coefficient and Jaccard index were also significantly correlated with the qualitative score.

**Table 4:** Correlation of the quantitative and qualitative concordance measures of original and updated structures.

	CTV Spearman correlation coefficient (p-value)	GTVp Spearman correlation coefficient (p-value)	GTVn Spearman correlation coefficient (p-value)
Dice Coefficient	0.06 (0.68)	0.46 (<0.01)	0.29 (0.15)
Jaccard index	0.07 (0.62)	0.45 (<0.01)	0.29 (0.14)
Mean Hausdorff (mm)	−0.22 (0.12)	−0.49 (<0.01)	−0.36 (0.06)
Sensitivity	0.56 (<0.01)	0.74 (<0.01)	0.06 (0.76)
Positive predictive value	−0.10 (0.49)	0.25 (0.07)	0.33 (0.09)

Twelve patients (22 %) were diagnosed with a locoregional recurrence. One patient was excluded from the recurrence-analyses as the recurrence could not be analyzed in relation to the original treatment due to salvage surgery performed after the completion of radiotherapy. In relation to the originally planned full-dose volume, 9 recurrences were in-field, 2 were marginal and none were out-of-field. The same was seen when treatment planning was reoptimised based on the new delineations with 9 recurrences in-field and 2 marginal. In relation to both the original and the new delineations, 9 recurrences were located fully inside the CTV, one was on the margin, and one was outside the CTV, thus no differences were seen between the original and the new delineations.

The 3-year and 5-year locoregional progression-free survival was 75.5 % (CI: 63.6–89.6 %) and 66.6 % (CI: 52.2–85.0 %), respectively. The median locoregional progression-free survival was not reached. The median overall survival was 18 months (range 3.9–153.8 months). The 3-year and 5-year overall survival was 40.7 % (CI: 29.5–56.2 %) and 29.6 % (CI: 19.6–44.7 %), respectively.

## 4 Discussion

The primary objective of this study was to analyze the impact of head and neck radiology expertise on the treatment volumes for hypopharyngeal tumours. In our cohort of 54 patients, we observed a significant reduction of the GTV volume when a radiologist was included in the delineation process.

We found low concordance between the original and the re-delineated GTV, indicating a significant impact of the collaboration with the radiologist. The reduction in GTV volumes could decrease high dose treatment volumes, and thereby potentially reduce side effects. Incorporating radiology expertise into target delineation may also reduce the risk of missing the target, as defining hypopharyngeal tumours on CT and MRI is challenging due to irregular growth and mucosal spread [13]. Previous studies have shown that radiological expertise influences definition of head and neck tumours, with changes to the GTV in over 50 % of evaluated cases, leading to a reduction in geographical misses [14,15]. These studies included few cases of hypopharyngeal cancers. However, Braunsetin et al found that 4 out of 5 hypopharyngeal cancer delineations were altered when reviewed by a radiologist.

We also observed a significant reduction in CTV volumes and a low concordance between the original and the re-delineated CTV. This reduction is only partly attributable to the decrease in GTV, as new departmental delineation guidelines, implemented after the treatment of patients in the current trial, also affected the re-delineated CTV. Similar to our findings, decreased CTV volumes were observed in a recent national Belgian project that introduced new delineation guidelines [16]. The reduction of CTV volumes may contribute to less side-effects and several studies have already shown the benefit of reducing elective CTV volumes [[17], [18], [19]]. It has also been demonstrated that reducing the CTV margin to the GTV can be safely performed, resulting in fewer side effects while maintaining the same level of locoregional control and survival [20].

Even though variations in the delineation of GTV and CTV are one of the greatest uncertainties in radiotherapy, the impact on tumour recurrence and survival remains unclear. However, a review of protocol compliance for the TROG 02.02 study in head and neck carcinoma demonstrated the critical importance of radiotherapy quality on survival and locoregional failure. One of the identified major quality deviations was incorrect definition of gross disease [21].

Theoretically, smaller target volumes increase the risk of locoregional failure. However, locoregional failure after radiotherapy for head and neck cancer is predominately located within the high-dose volumes [2,[22], [23], [24]]. This was also seen in our material, where no out-of-field relapses were noted. Furthermore, for the 11 patients with locoregional failure who could be evaluated, the median CTV volume after re-delineation was reduced to 44 % of the original volume, and all the recurrences were located within or adjacent to the re-delineated CTVs. Presumably, the reduction of the GTV- and CTV volumes noted in our results would not have increased the risk of loco-regional failure, and could potentially have led to fewer side-effects.

As the majority of recurrences in head and neck cancers occur in areas where high doses of radiotherapy have been administered, theoretically, intensifying the treatment is an appealing strategy to enhance the probability of tumour control. Due to the proximity of target volumes to organs at risk (OAR) in the head and neck region, alternative strategies to straightforward dose escalation have primarily been used. Accelerated radiotherapy regimens have demonstrated promising results; however, the addition of chemotherapy to these treatment protocols, which is standard practice for advanced tumors, has not resulted in significant clinical benefits [25]. Nevertheless, dose escalation has also been evaluated. Although, the randomized ArtDeco trial on modest dose escalation and hyperfractionation in patients with laryngeal or hypopharyngeal cancers, did not support this strategy, and the incidence of locoregional recurrence did not differ between the groups [26]. The lack of difference might, however, be attributed to the radiotherapy dosage, as patients in the dose-escalated arm received only 67.2 Gy in 28 fractions, compared to 65 Gy in 30 fractions in the standard-dose arm. Additionally, a study investigating the potential benefits of dose adaptation in squamous cell carcinomas of the head and neck, where dose escalation was guided by PET response, showed neither improved clinical outcomes nor an increased incidence of treatment-related toxicity [27].

Further research in this area is clearly needed. Our results indicate that the introduction of head and neck radiology expertise can reduce the GTV volumes. This, in combination with more advanced image guided radiotherapy techniques and adaptive strategies, could facilitate further dose escalation to the GTV without increasing doses to OARs and normal tissues in the pursuit of increased locoregional control. However, data is still lacking demonstrating a clear benefit of using adaptive radiotherapy in head and neck tumours [28]. This will probably change in the near future as new machines and software implementing automatic techniques reducing the resources needed for adaptive treatment strategies. Adaptive radiotherapy has thus the possibility of using precisely defined tumour and OAR structures to escalate the dose in the tumor while minimizing doses in healthy tissues [29].

There are some limitations to this study. The relatively small retrospective cohort makes it difficult to draw decisive conclusions. However, the tumour stages at diagnosis and the survival rates in our cohort are consistent with previously published data [30], suggesting that the selected patients are representative of a broader patient group. During the study period, new delineation guidelines were introduced, which complicates the interpretation of our data. However, the delineation guidelines relate only to CTV definition and do therefore not affect GTV delineation. Variations in target delineation related to the experience of the radiation oncologist may have introduced uncertainties and potential bias and re-delineation of the CTV from the original GTVs could have provided additional insights. However, given the retrospective nature of the study, we believe that comparing the original planned structures with the new structures based on updated GTVs provides a more practical and clinically relevant perspective. Furthermore, although variations in the expertise of delineating oncologists represent a significant factor, our routine practice of having senior oncologists peer-review contours helps mitigate this issue. Consequently, conclusions regarding differences in CTV delineation should be interpreted cautiously, as it is not feasible to independently assess the combined influences of radiologist support, evolving contouring guidelines, and variability in oncologist expertise. Nonetheless, to our knowledge, this is the first report on the possible benefit of radiological expertise in target delineation of hypopharyngeal tumours, in addition to peer-review by radiation oncologists. Therefore, despite the limitations mentioned above, it is a valuable contribution to the current knowledge on hypopharyngeal cancer, a disease with a dismal prognosis.

### 4.1 Conclusions

Our results indicate that incorporating radiological expertise in the target delineation of hypopharyngeal tumours reduces the defined tumour volumes and minimizes the risk of GTV overestimation. This may lead to a decrease in the irradiation of healthy tissues, thereby reducing side effects and improving patient outcomes.

## CRediT authorship contribution statement

**Gabriella Alexandersson von Döbeln:** Conceptualization, Data curation, Investigation, Methodology, Visualization, Writing – review & editing. **Eva Onjukka:** Data curation, Formal analysis, Validation, Visualization, Writing – review & editing. **Halla Sif Ólafsdóttir:** Visualization, Writing – review & editing. **Sara Jonmarker Jaraj:** Investigation, Writing – review & editing. **Mattias Hedman:** Conceptualization, Methodology, Project administration, Visualization, Writing – original draft, Writing – review & editing.

## Declaration of competing interest

The authors declare that they have no known competing financial interests or personal relationships that could have appeared to influence the work reported in this paper.
